# Antimicrobial Resistant *Salmonella* in Chelonians: Assessing Its Potential Risk in Zoological Institutions in Spain

**DOI:** 10.3390/vetsci9060264

**Published:** 2022-05-31

**Authors:** Clara Marin, Bárbara Martín-Maldonado, Marta Cerdà-Cuéllar, Sandra Sevilla-Navarro, Laura Lorenzo-Rebenaque, Laura Montoro-Dasi, Alicia Manzanares, Teresa Ayats, Aida Mencía-Gutiérrez, Jaume Jordá, Fernando González, Carlos Rojo-Solís, Carlos Barros, Daniel García-Párraga, Santiago Vega

**Affiliations:** 1 Facultad de Veterinaria, Instituto de Ciencias Biomédicas, Universidad Cardenal Herrera-CEU, CEU Universities, 46113 Alfara del Patriarca, Spain; s.sevilla@cecav.org (S.S.-N.); laura.lorenzorebenaque@uchceu.es (L.L.-R.); laura.montoro@uchceu.es (L.M.-D.); jaume.jorda@uchceu.es (J.J.); svega@uchceu.es (S.V.); 2 Grupo Estudio de la Medicina y Conservación de la Fauna Silvestre (GEMAS), 28220 Majadahonda, Spain; bmmjimenezvet@gmail.com (B.M.-M.); aidamengu@hotmail.com (A.M.-G.); fgonzalez@grefa.org (F.G.); 3 Grupo de Rehabilitación de la Fauna Autóctona y su Hábitat (GREFA), 28220 Majadahonda, Spain; 4 Deparment of Veterinary Medicine, School of Biomedical and Health Sciences, Universidad Europea de Madrid, 28670 Villaviciosa de Odón, Spain; 5 Unitat mixta d’Investigació IRTA-UAB en Sanitat Animal, Centre de Recerca en Sanitat Animal (CReSA), Campus de la Universitat Autònoma de Barcelona (UAB), 08193 Bellaterra, Spain; marta.cerda@irta.cat (M.C.-C.); alicia.manzanares@irta.cat (A.M.); teresa.ayats@irta.cat (T.A.); 6 IRTA, Programa de Sanitat Animal, Centre de Recerca en Sanitat Animal (CReSA), Campus de la Universitat Autònoma de Barcelona (UAB), 08193 Bellaterra, Spain; 7 Centro de Calidad Avícola y Alimentación Animal de la Comunidad Valenciana (CECAV), 12539 Les Alqueries, Spain; 8 Oceanogràfic Veterinary Services, Avanqua Oceanogàfic S.L., C/Eduardo Primo Yúfera (Científic) nº1B, 46013 Valencia, Spain; crojo@oceanografic.org (C.R.-S.); cbarros@oceanografic.org (C.B.); dgarcia@oceanografic.org (D.G.-P.)

**Keywords:** *Salmonella*, antibiotic resistance, zoonoses, tortoises, wildlife

## Abstract

*Salmonella* is mostly noted as a food-borne pathogen, but contact with chelonians has also been reported as a source of infection. Moreover, high levels of antimicrobial resistance (AMR) have been reported in *Salmonella* isolated from wild and captive reptiles. The aim of this study was to assess the occurrence of *Salmonella* AMR carriage by chelonians admitted to two zoological institutions in Spain, characterizing the isolates to assess the *Salmonella* AMR epidemiology in wildlife. To this end, 152 chelonians from nine species were sampled upon their arrival at the zoological nuclei. *Salmonella* identification was based on ISO 6579-1:2017 (Annex D), isolates were serotyped and their AMR analysed according to the EU Decision 2013/652. Moreover, the genetic relationship of the isolates was assessed by pulsed-field gel electrophoresis (PFGE). Results showed 19% (29/152) of the chelonians positive to *Salmonella*, all of them tortoises. For all isolates, 69% (20/29) were resistant and 34% (10/29) multidrug-resistant (MDR) strains. PFGE clustered isolates according to the serovar, confirming a low genetic diversity. In conclusion, this study shows a high presence of MDR *Salmonella* strains in tortoises at their entry into zoological nuclei. This condition highlights the need to establish *Salmonella* detection protocols for the entry of animals into these centres.

## 1. Introduction

*Salmonella* is considered one of the most important zoonotic agents with an estimated annual number of 93.8 million cases of salmonellosis worldwide [1,2]. In the European Union, salmonellosis was responsible for 87,923 human cases in 2019, of which 5.8% corresponded to Spain [3]. The infection usually causes a self-limited gastroenteritis, although some serovars can cause severe syndromes, such as Reiter’s Syndrome or Typhoid Fever, especially in children and elderly people, as they represent a risk population for this infection [4]. Despite the fact that *Salmonella* is mostly noted as a food-borne pathogen, the potential transmission of *Salmonella* from direct or indirect contact with reptiles cannot be ignored [5,6,7,8]. Indeed, reptile-associated salmonellosis (RAS), when the human *Salmonella* infection is acquired through contact with reptiles, is a growing public health concern worldwide [9]. Specifically, contact with turtles and tortoises has been widely associated with high risk of infection [10,11,12]. Indeed, several countries, such as the United States (US), have enacted a ban (40FR5620) to prevent chelonian-associated salmonellosis through the prohibition of turtles or turtle eggs with a carapace length of less than 10.16 cm; however, in Europe prevention is becoming more relevant [13,14,15,16,17]. Results from previous studies have shown a *Salmonella* incidence as high as 100% in free-living chelonians [18,19] and from 0% to 72.2% in pet chelonians [20,21]. *Salmonella* can be found in their intestinal tract and even in their environment. However, reptiles infected with *Salmonella* do not usually show any clinical signs. [12,21]. The close contact between chelonians and humans provides favourable conditions for the transmission of zoonotic infections, with reptile-associated salmonellosis being related to more severe clinical scenarios than those caused by other sources of infection [22,23,24].

Antimicrobial resistance (AMR) is one of the most important public health concerns and its control has become a goal in most countries [25]. In this sense, *Salmonella* has been included in the World Health Organisation priority list of twelve antibiotic-resistant bacteria [26]. Moreover, the development of multidrug-resistant (MDR) *Salmonella* strains could entail therapeutic consequences, with a complication in the treatment of both animals and humans [27,28]. High levels of AMR have been reported in *Salmonella* isolated from reptiles and there has been an increasing focus on the role of turtles as disseminators [24,29]. Thus, documented data revealed that about 100% and 73% of the *Salmonella* strains isolated from pet chelonians in Spain were AMR and MDR, respectively [22]. However, the incidence of MDR *Salmonella* in free-living chelonians in Spain is not well known. In this sense, wildlife rescue centres and zoos are places of entry for chelonians of various origins, where asymptomatic carriers could be vectors for the inter- and intra-specific transmission of resistant *Salmonella* within the zoological nucleus, and even to the staff [30,31].

In this context, the aim of this study was to assess the occurrence of AMR *Salmonella* carriage by chelonians admitted to two zoological facilities in Spain, characterising the isolates to gain more in-depth knowledge of *Salmonella* AMR epidemiology in wildlife.

## 2. Materials and Methods

All animals were handled according to the principles of animal care published by Spanish Royal Decree 53/2013 [32].

### 2.1. Sample Collection

For this study, different chelonians were sampled upon their arrival from captivity or from the wild at two different zoological nuclei. The first was the Wildlife and Habitat Rehabilitation Group (GREFA), a wildlife rescue centre located in Central Spain that admits almost 7000 wild animals yearly, including birds (raptors, such as Bonelli’s eagles (*Aquila fasciata*); waterfowl birds, such as mallards (*Anas platyrhynchos*); or passerines, such as Eurasian blue tits (*Cyanistes caeruleus*)), mammals (carnivores, such as red fox (*Vulpes vulpes*)); or ungulates, such as roe deer (*Capreolus capreolus*) and reptiles (lizards, such as ocellated lizard (*Timon lepidus*); chameleons, such as Mediterranean chamaleon (*Chamaleo chamaleon*); or snakes, such as the Montpellier snake (*Malpolon monspessulanus*)). All those animals belong to protected species of native Iberian fauna, and GREFA’s aim is to recover and release them back into the wild. The origin of most of the chelonians admitted to this centre is the captive breeding in private facilities or just illegal keeping. The second nucleus was the Oceanogràfic aquarium (OCE) located in the city of Valencia, on the Eastern coast of Spain. The OCE is a public zoological institution that aims to increase social awareness and public education to promote preservation of biodiversity. In addition to public display facilities, the OCE also supports regional government, providing veterinary support to the marine animal stranding network and acting as a rehabilitation centre for local marine fauna (including sea turtles) and with propagation programmes for local endangered species to be reintroduced back into the wild, including two chelonian species: Hermann’s tortoise (*Testudo hermanni*) and European pond turtle (*Emys orbicularis*). Finally, the OCE also serves as a holding facility for some confiscated allochthonous species or private owner donations.

From 2015 to 2019, all chelonians admitted to GREFA and OCE were sampled in order to assess their sanitary status before their accommodation at the zoological facilities. A total of 152 individuals were sampled in this study: tortoises (*n* = 81), pond turtles (*n* = 37) and sea turtles (*n* = 34). Overall, 77 individuals were sampled in GREFA and 75 in OCE. According to the origin of the animals, 84.6% from GREFA and 54.7% from OCE came from captivity; the rest of the animals were free-living individuals that were taken to these centres for their recovery (Table 1). Moreover, captivity Hermann’s tortoises from GREFA were donated by an owner that bred them yearly in his private garden, and all the European pond turtles were from the official GREFA´s captive breeding programme. Thus, all samples submitted by OCE came from tortoises from the same owner; where Aldabra, leopard and some radiated tortoises were kept together in one enclosure, with the rest of the radiated and all the marginated tortoises and other Hermann’s tortoises housed in a third facility. All sea turtle samples came from free living individuals.

From each individual, a cloacal swab was obtained using sterile cotton swabs during the first clinical examination. The swab cotton was inserted into the cloaca, and the swab was slowly twirling for 15 s to obtain the sample, and then kept in Cary–Blair transport medium (Cary–Blair sterile transport swabs, Deltalab^®^, Barcelona, Spain).

In tiny turtles, when the cloacal swab collection was not possible, each individual was housed singly in a plastic container with one litre of sterile water to prevent bacterial transmission. No filtration or antimicrobial treatment was added before sampling [19]. As bacteria excretion is not continuous, water samples were taken after two days in captivity. Then, 30 mL of water was taken and analysed. Negative control samples have been included in the analyses in order to detect possible contaminations.

Cloacal swabs and water samples were stored at 4 °C and processed for *Salmonella* detection within 24 h after collection.

### 2.2. Salmonella Detection and Serotyping

Samples were processed according to the ISO 6579-1:2017 (Annex D) recommendations for detection of *Salmonella* spp [33]. First, samples were pre-enriched in Buffered Peptone Water 2.5% (BPW; Scharlau^®^, Barcelona, Spain), in 1:10 *vol*/*vol* proportion, and incubated at 37 ± 1 °C for 18 ± 2 h. Then, pre-enriched samples were inoculated on a Modified Rappaport Vassiliadis agar plate (MSRV; Difco^®^, Valencia, Spain) and incubated at 41.5 ± 1 °C for 48 h. Colonies obtained on positive plates were transferred onto two specific agar plates for *Salmonella* spp. detection: Xylose-Lysine-Deoxycholate (XLD, Liofilchem^®^, Valencia, Spain) and a selective chromogenic medium (ASAP; bioMerieux^®^, Marcy l’Étoile, France). Both plates were incubated at 37 ± 1 °C for 24–48 h. A biochemical test (API-20E, bioMerieux, Marcy l’Étoile, France) was also performed to confirm *Salmonella*. Finally, *Salmonella* strains isolated were serotyped using the Kauffman–White scheme [34] and stored at −80 °C for further analysis.

### 2.3. Antimicrobial Susceptibility Testing

*Salmonella* strains were inoculated onto Müller–Hinton agar plates to perform the antimicrobial susceptibility test, based on the Kirby–Bauer disc diffusion method [35]. Antibiotics used for the test were those recommended by the 2013/652/EU document, including two quinolones: ciprofloxacin (CIP; 5 µg) and nalidixic acid (NA; 30 µg); one aminoglycoside: gentamicin (CN, 10 μg); one potentiated sulphonamide: trimethoprim-sulfamethoxazole (TMT-SXT; 25 µg); one phenicol: chloramphenicol (C; 30 µg); one pyrimidine: trimethoprim (TM; 5 µg); three b-lactams: ampicillin (AMP; 10 µg), cefotaxime (CTX; 30 µg) and ceftazidime (CAZ; 30 µg); one macrolide: azithromycin (AZM; 15 µg); one polymyxin: colistin (COL; 10 µg); and one glycylcycline: tigecycline (TGC; 15 µg) [36]. After 24 h of incubation at 37 °C, the inhibition zone around each disc was measured and interpreted according to the European Committee on Antimicrobial Susceptibility Testing (EUCAST) (http://www.eucast.org/clinical_breakpoints/, accessed on 22 July 2021) for *Enterobacteriaceae* and where this was not possible, according to Clinical and Laboratory Standards Institute (CLSI) indications (https://clsi.org/media/2663/m100ed29_sample.pdf, accessed on 22 July 2021) [22]. The isolates were classified as susceptible (S) or resistant (R) according to EUCAST Guidelines [37]. MDR was defined as acquired resistance to at least one agent in three or more antimicrobial classes [38].

### 2.4. Molecular Typing of Salmonella Isolates

Fresh bacterial cultures of *Salmonella* strains were prepared on Nutrient Agar (Oxoid Ltd., Madrid, Spain). The isolates were genotyped by pulsed-field gel electrophoresis (PFGE) according to the PulseNet standard operating procedure (www.pulsenetinternational.org, accessed on 22 July 2021). We performed the restriction enzyme digests with Xbal (Roche Applied Science, Indianapolis, IN, USA) and fragments were separated by electrophoresis in a CHEF-DR III System (Bio-Rad, Hercules, CA, USA). PFGE band patterns were analysed using Fingerprinting II software, v3.0 (Bio-Rad, Hercules, CA, USA). Cluster analysis was performed using the unweighted pair group method with arithmetic mean (UPGMA), using the Dice correlation coefficient with a band position tolerance of 1.5%. The isolates with a minimum level of similarity of 90% were considered genetically similar or identical and were assigned the same pulsotype.

### 2.5. Statistical analysis

A Generalised Linear Model, which assumed a binomial distribution for *Salmonella* shedding and AMR, was fitted to the data to determine whether there was an association with the categorical variables (chelonian species, and sample type). A reptile was considered *Salmonella*-positive if one of the samples collected (cloacal swabs or aquarium water samples) tested positive. A *p* ≤ 0.05 was considered to indicate a statistically significant difference. Data are presented as least squares means ± standard error of the least squares means. Analyses were carried out using a commercially available software application (SPSS 24.0 software package; SPSS Inc., Chicago, IL, USA, 2002).

## 3. Results

From all samples collected, 19.1% (29/152) tested positive for *Salmonella*. No significant differences were obtained between zoological nuclei (*p* > 0.05). However, all the positive samples were obtained from tortoises that arrived from captivity (36%; 29/81) (*p* < 0.001) (Table 2). Significant statistical differences for *Salmonella* isolation were found among the different tortoise species (*p* = 0.004), observing the highest percentage of positivity in Hermann’s tortoises (52.0%, 14/27) and marginated tortoise (40.0%, 8/20), followed by Aldabra giant tortoise and leopard tortoise (50.0%, 1/2, each), Greek tortoise (25.0%, 4/16), and finally radiated tortoise (7.0%, 1/14). None of the individuals presented symptomatology related to *Salmonella* infection.

From the 29 strains isolated, 28 *Salmonella* were identified as *Salmonella enterica* subsp *enterica* and one as *Salmonella enterica* subsp *salamae* (serovar 9,12:z29:1,5) (Table 3). The most represented serovar of *Salmonella enterica* subsp *enterica* was ser. Abony (37.0%, 10/27), followed by ser. Treforest (25.9%, 7/27), ser. Cerro and ser. Postdam (14.8%, 4/27, each), and finally ser. Warengo (11.1%, 3/27).

For all strains isolated, 69.0% (20/29) were resistant to at least one of the 12 antimicrobials tested. *Salmonella* strains isolated from marginated tortoise (*n* = 8), Aldabra giant tortoise, radiated tortoise, and leopard tortoise (*n* = 1, each) were AMR (Table 3). For Hermann’s tortoise and Greek tortoises, 50.0% of the isolated strains (7/14 and 2/4, respectively) were AMR. The highest percentages of AMR were found to CN (62.0%, *n* = 18), and CAZ (45.0%, *n* = 13), followed by TGC (34.0%, *n* = 10) and AZM (28.0%, *n* = 8), and finally AMP (3.0%, *n* = 1) (*p* < 0.05) (Table 3). Of the 12 antibiotics studied, no resistance was found against C, COL, CTX, NA, SXT, CIP, and TM.

**Table 3:** Antimicrobial resistance pattern of Salmonella strains.

Specie	Serovar	*n*	CIP	NA	CN	SXT	C	TM	AMP	CTX	CAZ	AZM	COL	TGC
*S. enterica* subsp *enterica*	Abony	10	0	0	2	0	0	0	0	0	2	0	0	0
	Postdam	4	0	0	1	0	0	0	0	0	0	0	0	0
	Treforest	7	0	0	7	0	0	0	0	0	5	5	0	6
	Cerro	4	0	0	4	0	0	0	0	0	4	1	0	2
	Warengo	3	0	0	3	0	0	0	1	0	1	1	0	2
*S. enterica* subsp *salamae*	9,12:z29:1,5	1	0	0	1	0	0	0	0	0	1	1	0	0

*n*: number of strains, CIP: ciprofloxacin, NA: nalidixic acid, CN: gentamicin, SXT: trimethoprim-sulphamethoxazole, C: chloramphenicol, TM: trimethoprim, AMP: ampicillin, CTX: cefotaxime, CAZ: ceftazidime, AZM: azithromycin, COL: colistin, TGC: tigecycline.

Furthermore, 34.5% (10/29) of *Salmonella* AMR isolates were considered MDR: ser. Cerro (50.0%, 2/4), ser. Treforest (71.4%, 5/7) and ser. Warengo (66.7%, 2/3). MDR *Salmonella* strains were isolated from marginated tortoises (100%, 7/7), Aldabra Giant tortoise (50%, 1/2), leopard tortoise (50%, 1/2) and Greek tortoise (6.2%, 1/16). Regarding the relationship among the serovar and MDR carriage, no association was observed (*p* = 0.777).

**Figure 1:**
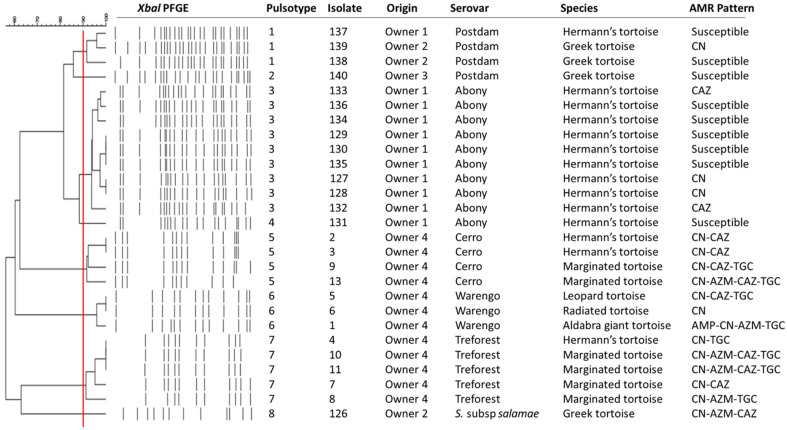
Dendrogram of the XbaI PGFE profiles from a subset of *Salmonella* strains isolated from chelonials. AMR: Antimicrobial resistance.

Overall, nine different AMR patterns were observed (Figure 1). The combination of CN-AZM-CAZ-TGC (25.0%, 5/20) was the most frequently observed, followed by CN alone (20.0%, 4/20) and CN-CAZ (15.0%, 3/20), CAZ alone and CAN-ACZ-TGC (10.0%, 2/20, each), and finally AMP-CN-AZM-TGC, CN-AZM-CAZ, CN-AZM-TGC, CN-TGC (5.0%, 1/20, each).

Of the 29 *Salmonella* isolates obtained in this study, 27 could be recovered for PFGE analysis. Two isolates could not be revived. A low genetic diversity was found with a total of eight different PFGE pulsotypes and isolates clustering according to their serovar; only two serovars ser. Abony and ser. Postdam showed two pulsotypes each, and the remaining serovars were represented by a single pulsotype (5, 6, 7 and 8, respectively) (Figure 1). The three most prevalent pulsotypes (3, 5 and 7) accounted for 66.7% of isolates (18/27) and 50% of serovars (3/6). The most frequent pulsotype (3) was found only in Hermann’s tortoise, while the second most frequent pulsotype (7) was isolated from Hermann’s tortoise and marginated tortoise. Hermann’s tortoise was the species with the highest diversity of *Salmonella* serovars and pulsotypes. The same AMR pattern was found among different pulsotypes and within each pulsotype.

## 4. Discussion

Historically, reptiles have been considered carriers of *Salmonella* spp. worldwide, which may pose a hazard as a source of environmental dissemination of the bacteria, while also being an important cause of animal and human infection [39,40,41]. Numerous studies have been conducted in pet reptiles, including chelonians, looking for different serovars of *Salmonella* that reptiles can host [21,42,43], but no studies have assessed the presence of *Salmonella* in chelonians admitted to wildlife rescue centres and zoos and the potential risk that can represent in releasable animals. The present study demonstrates that 36% of the tortoises examined upon arrival at the zoological facilities from private collections carry *Salmonella*. Nevertheless, *Salmonella* was not detected in pond turtles, sea turtles or other chelonians coming from the wild. Moreover, 69% of the strains isolated showed resistance to at least one antimicrobial, and 34% of them were MDR strains.

*Salmonella* has been isolated previously in 19% of the tortoises in households and pet shops from the Valencian Region, Spain [22]. This prevalence is lower than what was found in the present study (36%), highlighting the risks of the spread of the bacterium among tortoises housed in collectives, the risk of infection of animals from different species present in the facilities, and staff involved in rehabilitation practices and conservation projects [31]. It should be noted that the excretion of *Salmonella* by reptiles is extremely variable [44] and can be increased with stress, among other factors [9]. In this sense, prevalence obtained in the present study could be underestimated due to the intermittent shedding of *Salmonella*. In previous studies involving wild tortoises, the prevalence of *Salmonella* infection ranged from 34% to 100% [18,45,46]. Indeed, similarly to our study, a recent report of the incidence of *Salmonella* in captive tortoises in Italy revealed Hermann’s tortoise as the species in which the bacterium was most commonly isolated [31]. Moreover, none of the *Salmonella*-positive animals in our study had salmonellosis-related symptoms. Although clinical salmonellosis in reptiles is rare, it could occur, usually limited to intestinal signs or other symptoms such as dermatitis, salpingitis, septicaemia, osteomyelitis and granulomatous diseases, increasing the risk of transmission to humans [47,48].

No *Salmonella*-positive samples were found in free-living chelonians. Likewise, all the pond turtles and sea turtles were negative for *Salmonella*. Similarly, Strohl et al. [45] also reported no positive samples, while Hidalgo-Vila et al. [18] showed a low prevalence (12% and 15%) in the Mediterranean pond turtle (*Mauremys leprosa*) and the European pond turtle (*Emys orbicularis*), respectively. This difference with tortoises could be explained by the shorter time that *Salmonella* spends on the skin and in the cloaca in the aquatic animals. In terrestrial habitats, *Salmonella* persists for longer periods and is directly transmitted among individuals, favoured by the geophagy and coprophagy by tortoises, including faeces of feral animals such as birds or rodents [18,31,49]. Nevertheless, another hypothesis considered free-living reptiles as non-shedding carriers of *Salmonella*, excreting the bacteria only after long periods of stress [50]. Tortoises donated by private owners to both zoological nuclei had been kept in captivity for a long time, generally in high densities and in limited hygienic conditions. In contrast, free-living chelonians from nature have not, including sea turtles. On the other hand, European pond turtles were bred in captivity with all the proper biosecurity measures to ensure that those animals would not represent a potential risk to free-living populations when released.

In the present study, two subspecies of *S. enterica* belonging to seven different serovars were isolated (*S. enterica enterica* [I] and *S. enterica salamae* [II]). The most commonly reported serovars responsible for human salmonellosis, such as *S.* Enteritidis, *S.* Typhimurium, or monophasic *S.* Typhimurium, were not isolated in our study, similarly to other studies carried out in captive tortoises [18,31]. Nevertheless, the serovars isolated have been previously reported in human salmonellosis [18,41,46,51,52]. This includes ser. Abony, the most common in captive tortoises and wild tortoises in Spain [18,31,45], which has been associated with various cases of salmonellosis in infants and children [53], and in immunocompromised individuals [41,52], causing sepsis, meningitis, lung abscess and purulent pleuropneumonia [41,53]. *Salmonella* serovars Cerro, Postdam and Treforest have previously been isolated in chelonians in Spain, Italy and Taiwan [18,46,51], although is rarely associated with human disease [54,55]. To the author’s best knowledge, this is the first report of serovar Warengo in tortoises, and even in reptiles. *S. enterica* subsp *salamae* has been previously identified in turtles [18,56] and tortoises [18,46,49], but it is not associated with human infections [31].

From a One Health approach, where the environment, animals and humans are connected in a continuum, another epidemiological problem is the growing frequency of MDR strain threats [39]. A high percentage of *Salmonella* strains (69.0%) showed some AMR phenotype. Many studies have confirmed contamination of the environment with AMR bacteria [57] in soil [58], plants [59], and water [60]. In this context, *Salmonella*-positive tortoises could acquire AMR strains from contaminated soil or food and, of course, from previous antibiotic treatment.

The most frequent resistance pattern observed was CN-AZM-CAZ-TGC. CN-resistant *Salmonella* has been previously reported in tortoises, ranging from 1% to 23% [46,61]. In the present study, CN resistance was the most frequent. In this sense, the high prevalence of resistance has been observed previously in pet chelonians, with frequencies up to 100% [22]. CAZ is one of the first-line antimicrobial used in reptile medicine [62,63], commonly used for the treatment of salmonellosis in human and animals [64]. *Salmonella* isolates from the present survey showed a high percentage of resistance to CAZ, according to previous results [65], while most of the AMR studies carried out on chelonians show a high susceptibility to this antimicrobial [61,66]. To our best knowledge, this is the first report of AZM-resistant *Salmonella* detection in chelonians. AZM is one of the antimicrobials recommended by authorities to control salmonellosis in adults and children. Moreover, it represented the only option to treat extensively drug-resistant *Salmonella* Typhi in some regions of Asia before the emergence of AZM-resistant *Salmonella* [67]. TGC is another antimicrobial used against MDR bacteria. Detection of TGC-resistant *Salmonella* is rare [68], but Bertelloni et al. [66] observed 93.1% of isolates resistant to this antimicrobial in captive reptiles. Therefore, the control of MDR strains upon entry of new animals into wildlife nuclei must be very stringent to prevent their spread among individuals and the environment and to avoid future therapeutic failures [69].

Treforest has been reported as one of the most important serovars in human cases from the South-East Asian region [51]. While Hsu et al. [70] obtained only pansusceptible strains of this serovar from reptiles, Chen et al. [51] detected a high resistance prevalence to streptomycin among Treforest isolates. In our study, streptomycin was not analysed but instead, another aminoglycoside was included in the AMR test: CN. All the Treforest strains obtained in the present study showed a high frequency of AMR with 100% resistance to CN, 85.7% to TGC and 71.4% to both CAZ and AMZ.

The ability to link certain isolates to specific animals presented a unique opportunity to study *Salmonella* genetic diversity among chelonians. The PFGE typing showed the isolates were clustered according to the serovar, and a low genetic diversity within serovars was observed. It is important to remember the origin of animals: all the tortoises from OCE came from the same private owner, whereas captive animals from GREFA came from different sources (Figure 1). PFGE results could demonstrate the ability of the same strains to spread within the same population when there is close contact between individuals [22]. Finally, the higher *Salmonella* genetic diversity found in Hermann’s tortoise is also related to the origin of the animals, as this is the only species that was admitted at both zoological facilities.

## 5. Conclusions

The characterising of the isolates obtained from chelonians showed that only tortoises were positive for *Salmonella* spp., being Abony the main serovar isolated. Moreover, a high presence of MDR *Salmonella* strains was found at the individual’s arrival into zoological nuclei, with, a strong genetic relationship between the 66.7% of the strains isolated. These facts highlight the importance of establishing strict *Salmonella* detection protocols upon the arrival of new animals at a zoological nucleus to prevent the spread of resistant bacteria to other resident animals or to the workers.

## Figures and Tables

**Table 1:** Species, origin details of chelonians sampled in this study.

Zoological Nucleus	Chelonian Species	*n*	Origin	
			Captivity	Nature
GREFA	Greek tortoises (*Testudo graeca*)	16	16	0
	Hermann’s tortoises (*Testudo hermanni*)	24	22	2
	European pond turtles (*Emys orbicularis*)	18	18	0
	Mediterranean pond turtles (*Mauremys leprosa*)	19	10	9
OCE	Marginated tortoise (*Testudo marginata*)	20	20	0
	Hermann’s tortoises (*Testudo hermanni*)	3	3	3
	Radiated tortoise (*Astrochelys radiata*)	14	14	0
	Aldabra giant tortoise (*Aldabrachelys gigantea*)	2	2	0
	Leopard tortoise (*Stigmochelys pardalis*)	2	2	0
	Loggerhead sea turtle (*Caretta caretta*)	34	0	34

GREFA: Wildlife and Habitat Rehabilitation Group; OCE: Oceanogràfic; *n*: number of individuals.

**Table 2:** Details of *Salmonella* detection among the different chelonian species.

Zoological Nucleus	Chelonian Type	Chelonian Species	*n*	*Salmonella*-Positive (%)
GREFA	Tortoise	Greek tortoise (*Testudo graeca*)	16	25
		Hermann’s tortoise (*Testudo hermanni*)	24	45.8
	Pond Turtle	European pond turtle (*Emys orbicularis*)	18	0
		Mediterranean pond turtle (*Mauremys leprosa*)	19	0
OCE	Tortoise	Marginated tortoise (*Testudo marginata*)	20	40
		Hermann’s tortoises (*Testudo hermanni*)	3	100
		Radiated tortoise (*Astrochelys radiata*)	14	100
		Aldabra giant tortoise (*Aldabrachelys gigantea*)	2	50
		Leopard tortoise (*Stigmochelys pardalis*)	2	50
	Sea Turtles	Loggerhead sea turtle (*Caretta caretta*)	34	0

*n*: number of individuals. GREFA: Wildlife and Habitat Rehabilitation Group; OCE: Oceanogràfic.

## Data Availability

Data is available upon reasonable request.
